# Overcoming the difficulties of predicting conformational polymorph energetics in molecular crystals *via* correlated wavefunction methods†
†Electronic supplementary information (ESI) available: Additional computational details, tables of energies, analysis of model convergence, & optimized crystal structures. See DOI: 10.1039/c9sc05689k


**DOI:** 10.1039/c9sc05689k

**Published:** 2020-01-14

**Authors:** Chandler Greenwell, Jessica L. McKinley, Peiyu Zhang, Qun Zeng, Guangxu Sun, Bochen Li, Shuhao Wen, Gregory J. O. Beran

**Affiliations:** a Department of Chemistry , University of California , Riverside , California 92521 , USA . Email: gregory.beran@ucr.edu ; Tel: +1-951-827-7869; b Xtalpi, Inc. , 245 Main St, 12th Floor , Cambridge , MA 02142 , USA

## Abstract

Molecular crystal structure prediction is increasingly being applied to study the solid form landscapes of larger, more flexible pharmaceutical molecules. Despite many successes in crystal structure prediction, van der Waals-inclusive density functional theory (DFT) methods exhibit serious failures predicting the polymorph stabilities for a number of systems exhibiting conformational polymorphism, where changes in intramolecular conformation lead to different intermolecular crystal packings. Here, the stabilities of the conformational polymorphs of *o*-acetamidobenzamide, ROY, and oxalyl dihydrazide are examined in detail. DFT functionals that have previously been very successful in crystal structure prediction perform poorly in all three systems, due primarily to the poor intramolecular conformational energies, but also due to the intermolecular description in oxalyl dihydrazide. In all three cases, a fragment-based dispersion-corrected second-order Møller–Plesset perturbation theory (MP2D) treatment of the crystals overcomes these difficulties and predicts conformational polymorph stabilities in good agreement with experiment. These results highlight the need for methods which go beyond current-generation DFT functionals to make crystal polymorph stability predictions truly reliable.

## Abstract 2

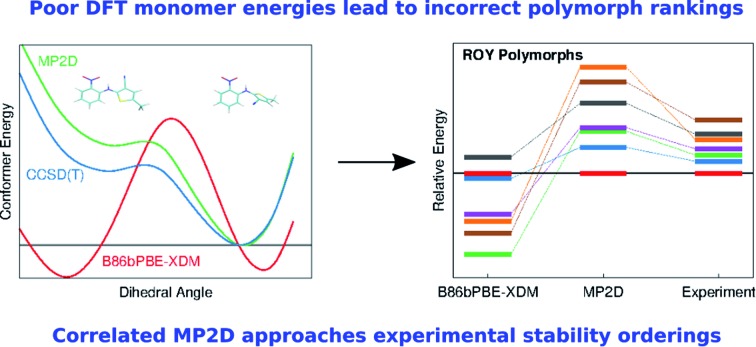
Widely used crystal structure prediction models based on density functional theory can perform poorly for conformational polymorphs, but a new model corrects those polymorph stability rankings.

## 1 Introduction

Crystal packing influences the physical properties of organic crystals. The occurrence of multiple crystalline packing motifs, or polymorphs, of a pharmaceutical can impact its solubility, bioavailability, shelf-life/stability, and tabletting properties, for example. The importance of polymorphism to the pharmaceutical industry is highlighted by examples such as ritonavir^1^,^2^ and rotigotine,^3^ where the late-stage appearance of more stable, less soluble crystal forms forced product recalls and reformulations. It was recently suggested that the thermodynamically stable crystal form has not been realized experimentally for ∼15–45% of pharmaceutical molecules,^4^ raising speculation that more such examples may occur in the future. Moreover, solid form patents play an important role in the commercial life cycle of a drug, as evidenced by the recent legal wrangling over a new polymorph of Celgene's blockbuster drug Revlimid that was discovered by generic drug manufacturer Natco.^5^

The ability to predict the molecular crystal energy landscape, which is the set of possible low-energy crystal structures for a given compound, would be a tremendous boon to the pharmaceutical industry and others. Crystal structure prediction has long been challenging^6^ due to the complexity of the search space, the small energy differences that separate polymorphs, and the complexities of crystallization kinetics. The accuracy requirements for predicting the crystal energy landscape are severe: surveys suggest that about half of all polymorph pairs are separated by less than 2 kJ mol^–1^ in lattice energy, and around 95% are separated by less than 8 kJ mol^–1^.^7^–^9^

The advent of high-quality, dispersion-corrected density functional theory (DFT) models^10^–^13^ has enabled tremendous progress in the energy ranking aspects of crystal structure prediction, as evidenced by results from the recent blind tests^14^–^17^ and other studies.^18^–^30^ Increasingly, DFT is being called on to explore pharmaceutical crystal energy landscapes as a complement to experimental solid form screening.^31^–^39^ Computational prediction of a highly stable, unrealized polymorph of galunisertib played a key role in the extensive characterization of its solid form landscape, for example.^38^

Despite many successes of DFT-driven crystal structure prediction, close inspection of the literature also finds polymorphic crystals for which widely-used DFT models fail dramatically. Many of these difficult cases involve conformational polymorphs, in which different intramolecular conformations enable different intermolecular crystal packing motifs. For example, DFT methods invert the polymorph stability ordering of α and β *o*-acetamidobenzamide, with errors of 5–10 kJ mol^–1^.^40^ The prolific polymorph-former 5-methyl-2-[(2-nitrophenyl)amino]-3-thiophenecarbonitrile, nicknamed “ROY” after its colorful red-orange-yellow crystals, is another example. State-of-the-art density functional models predict the Y polymorph to be one of the least stable forms,^41^,^42^ when it is actually the most stable one. These backwards stability rankings reflect errors approaching 10 kJ mol^–1^. In another case, crystal structure prediction failed for two of six conformationally flexible species resulting from mechanochemical aromatic disulfide metathesis reactions, with errors exceeding 6 kJ mol^–1^ due in large part to poor intramolecular DFT conformational energies.^43^ Erroneous intramolecular conformational energies caused a similar failure for a recent DFT study of Molecule X from an earlier blind test of crystal structure prediction.^24^

The large errors in the relative polymorph stabilities found for many of these examples greatly exceed the few kJ mol^–1^ errors or less one typically finds for DFT in successful crystal structure prediction cases. Furthermore, these errors are catastrophically large compared to the small energy differences that are characteristic of polymorphism. The pharmaceutical industry trend toward developing larger, more flexible drug molecules^44^ makes problems with ranking conformational polymorphs particularly concerning, since it raises the possibility that such ranking problems will become more prevalent as crystal structure prediction is applied to increasingly complicated species.

The problems with popular DFT functionals are not limited to conformational polymorphism either. Delocalization error in commonly used DFT generalized gradient approximation (GGA) functionals can cause spurious salt formation in co-crystals^45^ and the substantial overbinding of crystals containing halogen bonds.^46^ Many functionals erroneously predict the exothermic anthracene photodimerization reaction to be strongly endothermic,^47^,^48^ which is problematic^49^ when studying a class of interesting anthracene-based photomechanical materials.^50^,^51^

Switching to a hybrid density functional can address some of the limitations of GGA-type functionals that cause incorrect polymorph rankings and other problems,^20^,^28^,^45^,^46^,^52^,^53^ but it does not rectify the incorrect ROY polymorph rankings,^41^ for example. Approaches based on periodic second-order Møller–Plesset perturbation theory (MP2),^54^–^68^ the random phase approximation (RPA),^68^–^71^ and quantum Monte Carlo^72^–^75^ are also being developed that can improve the reliability of polymorph stability rankings. Computational cost is the fundamental challenge that inhibits applying these more accurate electronic structure methods to molecular crystals. As evident from the studies cited above, higher-level electronic structure methods have generally been applied only to small-molecule crystals at present.

Fragment-based methods provide one means of lowering the computational cost of correlated electronic structure methods by decomposing the molecular crystal into monomers, dimers, and many-body contributions.^10^,^76^–^80^ These methods typically compute only the key monomer and dimer contributions at the most accurate level of theory, while the many-body contributions are approximated in some fashion. Employing coupled cluster singles, doubles, and perturbative triples (CCSD(T)) in the context of a fragment method can give very reliable results, as demonstrated by quantitative prediction of the benzene lattice energy^81^ or the prediction of the methanol polymorph phase diagram with ∼0.5 kJ mol^–1^ accuracy.^82^

Unfortunately, even with fragment methods, CCSD(T) calculations are cost-prohibitive for crystals involving pharmaceutical-sized species. MP2 is more feasible computationally, but it suffers from well-known problems in the description of van der Waals interactions^83^ that cause it to substantially overestimate the interaction energy in π-stacking complexes^84^ and to over-bind the benzene crystal by ∼10–20%,^10^ for example. This difficulty is overcome here by using the recently developed MP2D model, which employs a Grimme D3-like^85^ dispersion correction that removes the problematic dispersion treatment inherent to MP2 and replaces it with a more reliable treatment. MP2D performs well across extensive benchmark calculations of dimer interactions, molecular conformations, and reaction energies,^48^ and it correctly describes the energetics of the aforementioned anthracene photodimer.^49^

**Fig. 1:**
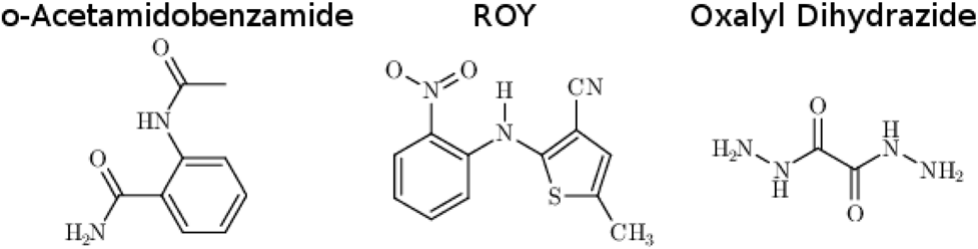
The species whose conformational polymorphs are studied here.

The present study examines three challenging cases of conformational polymorphism in detail: *ortho*-acetamidobenzamide,^40^ ROY,^42^,^86^ and oxalyl dihydrazide (Fig. 1).^40^,^87^ It demonstrates how problems in the intramolecular conformational energies and, to a lesser extent, the intermolecular interactions with well-regarded dispersion-corrected DFT functionals lead to incorrect polymorph stabilities. However, modeling these systems with fragment-based correlated wavefunction methods overcomes these difficulties, restoring the crucial balance between intra- and intermolecular interactions^88^–^90^ that is required to predict the correct stabilities in conformational polymorphs. The results here highlight how despite considerable progress with DFT, polymorph stability ranking remains challenging, and models that can achieve higher accuracy than that of commonly used DFT approximations are needed before polymorph ranking can be considered a “solved” problem.

## 2 Theory and methods

When predicting relative polymorph stabilities, it is important to recognize that those stabilities depend on temperature. Sometimes the free energy variations are large enough to produce an enantiotropic relationship where the thermodynamically preferred polymorph changes depending on the temperature. However, even in monotropic cases where one polymorph is always preferred thermodynamically, the magnitude of the enthalpy and free energy differences between two polymorphs will depend on temperature. The temperature dependence of the relative stabilities arises from both phonon contributions to the vibrational partition function and from phonon-driven thermal expansion. The molar volume of typical organic crystals expands several percent upon heating from 0 K to room temperature.^91^ This expansion alters the lattice energy and introduces anharmonicity into the phonons. Accounting for it is important for quantitatively comparing thermochemistry,^82^,^92^–^94^ mechanical properties,^93^ and spectroscopic observables^91^,^95^ between theory and experiment.

It is therefore important to consider the thermodynamic conditions under which experimental measurements were made when making theoretical predictions. Ideally, one would capture temperature effects *via* molecular dynamics (including nuclear quantum effects, since zero-point contributions can be significant^94^,^96^). However, molecular dynamics simulations based on high-level electronic structure methods are very computationally expensive.^96^ The quasi-harmonic approximation is often successfully used to approximate the volume-dependent contributions to the phonons and lattice energies.^13^,^23^,^28^,^82^,^91^–^94^,^97^–^100^ Nevertheless, quasi-harmonic calculations remain considerably more expensive than purely harmonic calculations that neglect thermal expansion.

Here, a simple approximation is employed to estimate the temperature dependence of the thermochemical stabilities and facilitate comparison with experiment. Two types of crystal structure optimizations are performed. Fully relaxed crystal structures that optimize both the atomic positions and the unit cell vectors approximate the structure at 0 K (albeit without zero-point vibrational expansion^94^). Room-temperature structures are mimicked *via* fixed-cell optimizations that relax the atomic positions subject to the constraint of the room-temperature experimental lattice parameters. Harmonic phonons are computed separately on each set of structures, thereby approximately capturing the anharmonicity that results from the change in unit cell dimensions.

Similar fixed-cell optimizations have been used by many other authors previously to examine room-temperature crystal properties, including in earlier studies on the same polymorphic systems studied here.^40^,^42^ Constraining the lattice parameters effectively captures the thermal expansion effects and its associated phonon anharmonicity, while relaxing the atomic positions addresses any issues in the experimental molecular geometries (hydrogen atom placement, for example) and ensures the structure is at a minimum for harmonic vibrational frequency calculations. Additional support for this approach comes from the fact that nuclear magnetic resonance chemical shift predictions performed on structures relaxed with fixed lattice parameters reproduce experimental chemical shifts better than those obtained from fully relaxed structures^91^ or even neutron diffraction structures.^101^

Note that the approximations used here neglect thermal/large-amplitude dynamical motions that can occur in molecular crystals. Fortunately, the structures of the systems considered here do not exhibit significant disorder and are likely amenable to static modeling treatments. The differences between quasi-harmonic and molecular dynamics models are frequently (but not always) small.^102^–^104^ In the end, combining information from the fully-relaxed 0 K structures and fixed-cell room-temperature structures provides information regarding the topology of crystal energy landscapes that facilitates comparison with experiment.

Experimental crystal structures were obtained from the Cambridge Structure Database for *o*-acetamidobenzamide^105^ (reference codes ACBNZA and ACBZNA01), ROY^106^–^108^ (QAXMEH–QAXMEH05, QAXMEH12, and QAXMEH52), and oxalyl dihydrazide^87^ (VIPKIO01–VIPKIO05). Crystal structures were optimized using periodic DFT with the B86bPBE density functional^109^,^110^ and exchange-hole dipole moment (XDM) dispersion correction.^111^ This particular combination performs well in many molecular crystal applications.^22^–^24^,^111^

Single-point refinement of the electronic energies was carried out using correlated wavefunction methods *via* the fragment-based hybrid many-body interaction (HMBI) model.^78^,^112^–^114^ HMBI partitions the total energy of the crystal into intramolecular contributions (1-body interactions), pairwise intermolecular interactions (2-body interactions), and the remaining many-body intermolecular lattice contributions. The important 1-body and short-range (SR) 2-body terms are modeled with high-level electronic structure methods (*e.g.* CCSD(T) or MP2-based methods here), while the longer-range (LR) 2-body and many-body contributions are modeled with periodic Hartree–Fock (HF) theory (which makes it comparable to Stoll's method of increments^76^).

1 *U*HMBIel = *E*High1-body + *E*HighSR 2-body + *E*HFLR 2-body + *E*HFmany-body

As noted above, MP2 suffers from problematic description of van der Waals interactions. The related and highly successful^115^,^116^ MP2C model addresses this problem by adding a non-empirical intermolecular dispersion correction to MP2.^117^,^118^ However, the MP2C correction is derived from intermolecular perturbation theory and does not address problems with intramolecular dispersion. MP2D^48^ expresses the dispersion correction in terms of atom-centered *C*_6_ and *C*_8_ dispersion coefficients^119^ computed using the scheme behind Grimme's D3 dispersion correction.^85^ The five global empirical parameters in MP2D were determined previously^48^ on small-molecule systems that do not include the species studied here.

In the cases of oxalyl dihydrazide and *o*-acetamidobenzamide, enthalpies and free energies are computed for comparison with experiment. This requires evaluating harmonic phonon contributions to the enthalpy and Helmholtz vibrational free energy *F*_vib_*via* the standard statistical mechanical expressions.^94^ The phonons and their thermodynamic contributions are calculated at the B86bPBE-XDM level, using either the 0 K or room-temperature crystal structures, and these are used to augment the electronic energy computed with either DFT or HMBI. For example, the Gibbs free energy at the MP2D level is estimated as,

2 *G*(*T*,*P*) = *U*HMBIel + *F*DFTvib + *PV*

For a crystal at ambient conditions, the *PV* term contributes negligibly and can be ignored. This combination of DFT geometries and phonons with higher-level single-point electronic energies has been validated previously.^116^

The DFT calculations were performed using Quantum Espresso v6.3 (ref. 120) using a 50 Ry planewave cutoff and well-converged Monkhorst–Pack *k*-point sampling grids (ESI Section S1.1†). Core electrons were treated according to the projector augmented wave (PAW) approach using PAW potentials for H, C, N, O, and S produced with A. Dal Corso's Atomic code v6.1.^121^ Gas-phase monomer and dimer DFT calculations used in the energy decompositions were performed in large unit cells with a minimum of 15 Å spacing between the central monomer/dimer atoms and all periodic image atoms. Using an even larger 18 Å spacing altered the gas-phase energies by ∼0.15 kJ mol^–1^ or less, indicating that this vacuum spacing is appropriately large to mimic the gas phase.

Harmonic DFT phonon frequencies for oxalyl dihydrazide and *o*-acetamidobenzamide were computed at the *Γ*-point using Phonopy v1.12.6-r66 (ref. 122) with the same B86bPBE-XDM functional and basis set used for the energies and geometry optimizations. To ensure equal numbers of molecules (*Z* = 4) in the cell for each of the five oxalyl dihydrazide polymorphs, supercells were constructed for the α, β, δ, and ε forms by doubling the cell along the shortest crystallographic axis. Using larger supercells and/or capturing phonon dispersion away from the *Γ* point would certainly improve the quality of the predicted thermochemistry.^100^ Still, earlier quasi-harmonic sublimation enthalpy calculations for several small-molecule crystals agreed with experiment to within a couple kJ mol^–1^ despite neglecting phonon dispersion.^116^ Percentage errors in the entropic contributions were considerably larger for the same species, however. Phonons were not computed for the larger ROY system for reasons of computational expense.

For the HMBI fragment calculations employing correlated wave function methods, large basis sets must be used to ensure convergence of the polymorph energetics, as demonstrated in ESI Section S1.2† and many previous studies.^10^,^81^,^82^,^93^,^94^,^116^,^123^,^124^ Here, MP2 and MP2D monomer and dimer energies at the complete-basis-set (CBS) limit were obtained using a development version of PSI4.^125^ The correlation energy was extrapolated^126^ to the complete-basis-set (CBS) limit using data from the aug-cc-pVTZ and aug-cc-pVQZ basis sets^127^ and combined with HF/aug-cc-pVQZ. The MP2C dispersion corrections were obtained with Molpro 2012.1 (ref. 128) in the aug-cc-pVTZ basis set. The MP2C dispersion correction typically converges faster with basis set than the raw correlation energy.^118^ CCSD(T) results at the CBS limit were obtained by correcting MP2/CBS energies with the difference between CCSD(T) and MP2 in the aug-cc-pVDZ (ROY, oxalyl dihydrazide) or cc-pVTZ (acetamidobenzamide intramolecular contributions) basis sets. The periodic HF many-body contributions were evaluated using Crystal 17 (ref. 129) and the pob-TZVP-rev2 basis set.^130^ This basis set was chosen based on cluster benchmarks described in ESI Section S1.3.† Note that due to computational expense, large-basis set calculations were performed only on the room-temperature structures of ROY, since those are more directly comparable with experiment. Smaller-basis results on the fully relaxed structures are provided in ESI Section S3.3.† As expected, large basis sets are required to converge the relative polymorph stabilities.

As part of the analysis of the ROY system, a one-dimensional, gas-phase conformational energy scan over the key S–C–N–C dihedral angle was performed. At each of 16 fixed dihedral angle values ranging 0–150° in 10° intervals, all other degrees of freedom were fully relaxed at the B3LYP-D3(BJ)/def2-TZVP level of theory. Single-point energies were then computed on these geometries using B86bPBE-XDM, MP2, MP2D, and CCSD(T).

## 3 Results and discussion

### 3.1 *o*-Acetamidobenzamide

**Fig. 2:**
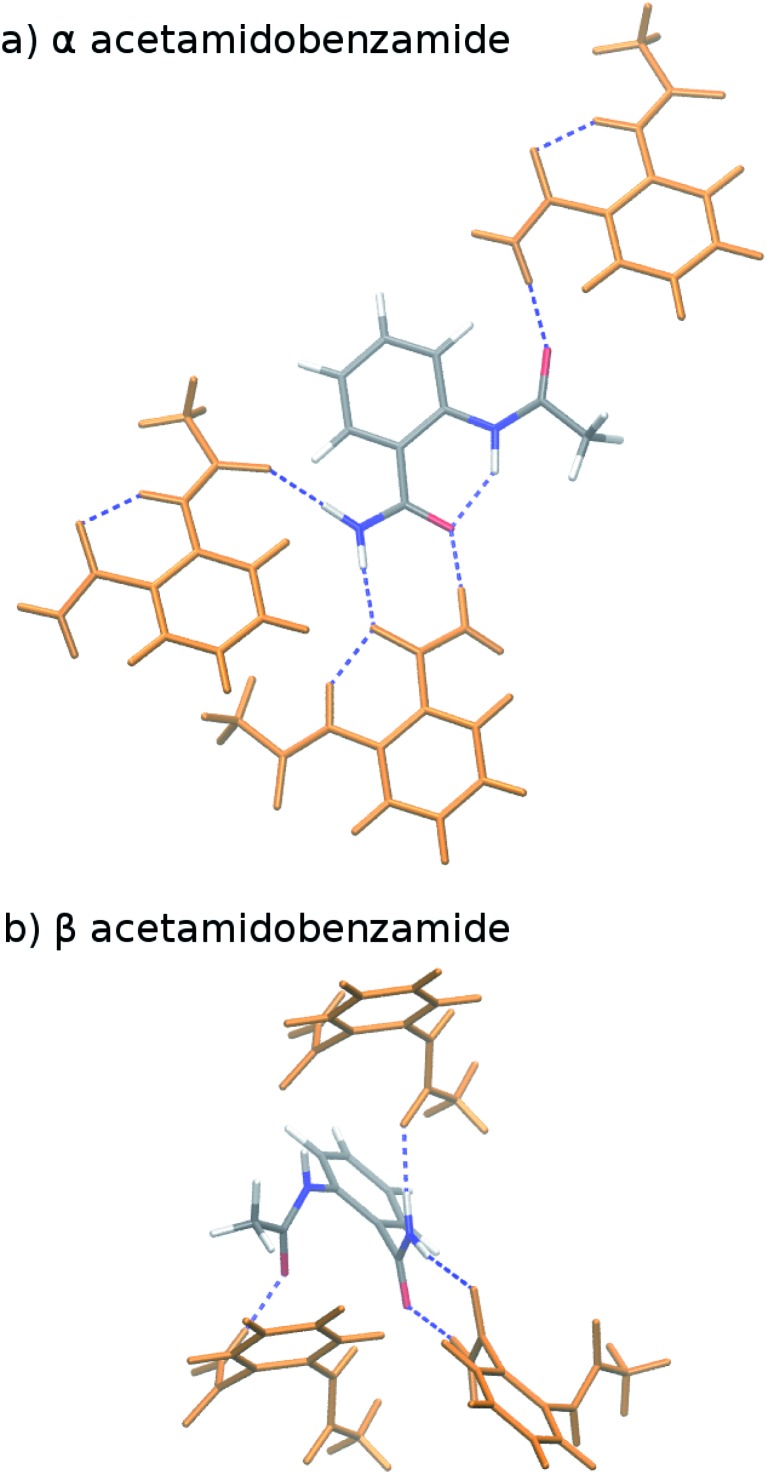
Local hydrogen bonding environments for crystalline *o*-acetamidobenzamide. (a) In the α polymorph, the molecule is nearly planar and adopts an intramolecular hydrogen bond, while (b) in the β polymorph the amide and acetamide side chains rotate out of the plane to achieve better intermolecular hydrogen bonds.

*o*-Acetamidobenzamide has two conformational polymorphs: the α form adopts the more stable intramolecular conformation containing an intramolecular hydrogen bond between the acetamide hydrogen and the amide oxygen (Fig. 2).^105^ The β polymorph sacrifices the intramolecular hydrogen bond to adopt a conformation that allows better intermolecular hydrogen bonding. Experimentally, the α form converts exothermically and irreversibly to β upon heating to 150 °C (423 K), with Δ*H*_α→β_ values of –1.9 kJ mol^–1^ (ref. 40) or –2.9 kJ mol^–1^ (ref. 105). In other words, the β form is clearly preferred over the α one at high temperatures, though the stability ordering at lower temperatures is unclear.

**Fig. 3:**
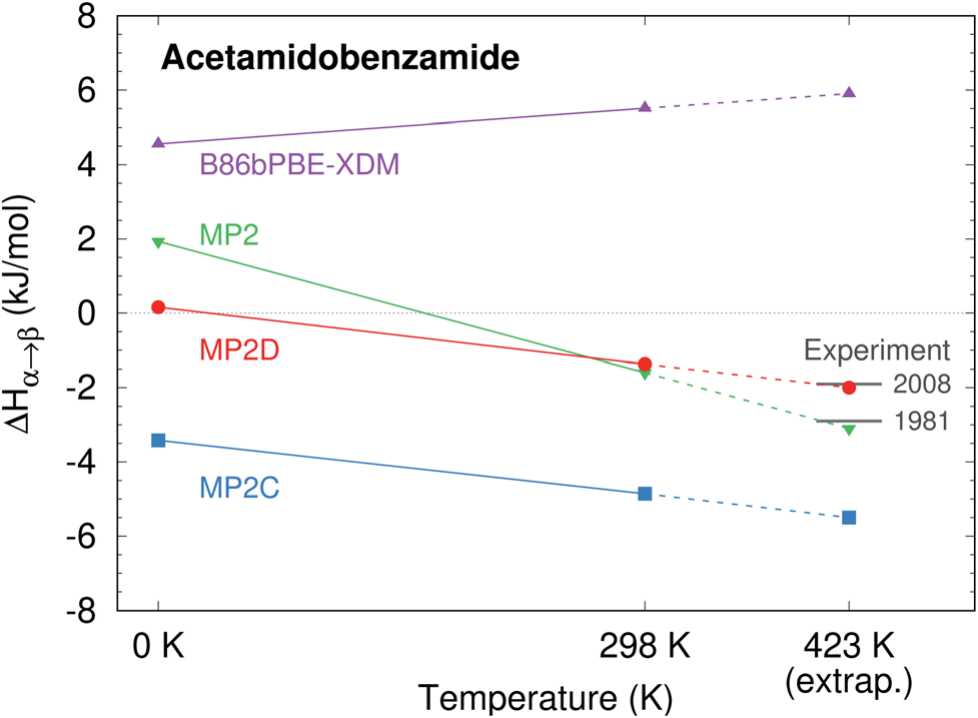
Predicted enthalpy difference between the α and β polymorphs of *o*-acetamidobenzamide at 0 K, room temperature, and linearly extrapolated to 423 K. Experimental values were taken from ref. 40 and 105.

Earlier calculations using force fields and several different GGA and hybrid DFT functionals all predict the α form lattice energy to be ∼5–10 kJ mol^–1^ more stable than β.^40^ New B86bPBE-XDM DFT lattice energy calculations performed here similarly favor the α form by 5.8 kJ mol^–1^ (Table 1). The B86bPBE-XDM harmonic zero-point vibrational energy contribution for the fully-relaxed 0 K structures stabilizes the β form by 1.2 kJ mol^–1^ relative to α, which is reasonably similar to the 2 kJ mol^–1^ value estimated previously.^40^ In other words, the difference in zero-point energy contributions between polymorphs are much too small to alter the B86bPBE-XDM polymorph stability ordering. Moreover, the DFT enthalpic preference for the α form increases from 4.6 kJ mol^–1^ at 0 K to 5.5 kJ mol^–1^ at room temperature (Fig. 3). As shown in ESI Table S4,† this temperature-dependence between the 0 K and room-temperature structures arises from a 0.6 kJ mol^–1^ relative destabilization of the α form caused by the lattice energy changes that is canceled by a larger 1.6 kJ mol^–1^ stabilization due to the vibrational enthalpy contribution.

**Table 1:** Stability of the β *o*-acetamidobenzamide polymorph relative to the α one, in kJ mol^–1^. Positive values indicate α is more stable than β. See ESI Table S4 for additional details of the lattice energy and phonon contributions

Method	Δ*E*_intra_(0 K)	Δ*E*_inter_(0 K)	Δ*E*_lattice_(0 K)	Δ*H*(0 K)	Δ*H*(298 K)	Δ*H*(423 K)^*a*^	Δ*G*(298 K)
B86bPBE-XDM	58.0	–52.2	5.8	4.6	5.5	5.9	4.3
MP2/CBS + pHF	50.1	–46.8	3.2	1.9	–1.6	–3.1	–2.8
MP2C/CBS + pHF	50.1	–52.2	–2.1	–3.4	–4.9	–5.5	–6.1
MP2D/CBS + pHF	52.6	–51.2	1.4	0.2	–1.4	–2.0	–2.6
CCSD(T)/CBS	52.3						
Experiment						–1.9^*b*^, –2.9^*c*^	

^*a*^Linearly extrapolated to 423 K from Δ*H*(0 K) and Δ*H*(298 K) values.

^*b*^ Ref. 40.

^*c*^ Ref. 105.

**Fig. 4:**
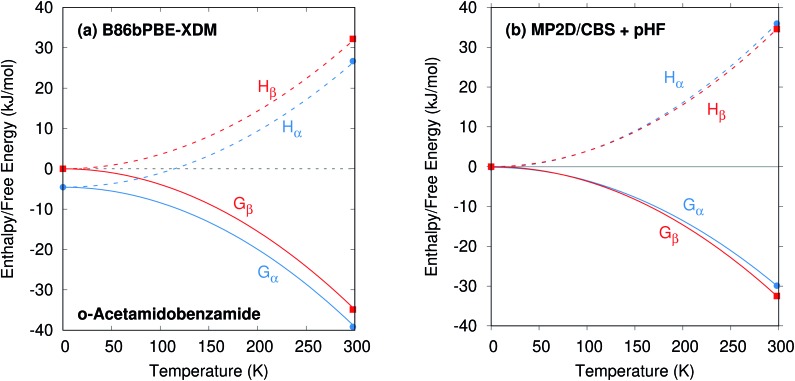
Schematic relative enthalpy *H* and free energy *G* curves for the α and β polymorphs of *o*-acetamidobenzamide computed using (a) B86bPBE-XDM or (b) fragment-based MP2D/CBS + pHF. The MP2D model reverses the stability ordering, giving results that are consistent with experiment.

To compare more directly against experiment, the transition enthalpy at the phase transition temperature is estimated here *via* linear extrapolation of the 0 K and room-temperature results. The resulting endothermic Δ*H*_α→β_(423 K) value of 5.9 kJ mol^–1^ contradicts the exothermic phase transition observed experimentally. The B86bPBE-XDM Gibbs free energies exhibit a clear preference for the α polymorph of 4.6 kJ mol^–1^ at 0 K that decreases only slightly to 4.3 kJ mol^–1^ at room temperature (Table 1). Linear extrapolation of Δ*G*_α→β_ to 423 K gives 4.2 kJ mol^–1^. This strong DFT free energy preference for the α form is inconsistent with the irreversible α → β phase transition seen experimentally. Earlier attempts to rationalize the DFT lattice energy preference for the α polymorph suggested that the two polymorphs might be enantiotropically related.^40^ However, the DFT harmonic free energy calculations here predict the α polymorph to be considerably more stable throughout the temperature range (*i.e.* monotropically related, see Fig. 4a). Taken together, both the B86bPBE-XDM enthalpies and free energies computed here and those reported previously^40^ are inconsistent with experiment.

In contrast, fragment-based MP2D predicts polymorph stabilities that agree very well with experiment (Table 1). Calculations on the fully-relaxed 0 K structures suggest that the α polymorph is still more stable in lattice energy, but by a much smaller 1.4 kJ mol^–1^. Including the B86bPBE-XDM zero-point vibrational contribution preferentially stabilizes the β form, such that the two forms become nearly degenerate (α is 0.2 kJ mol^–1^ more stable than β). Heating further stabilizes the β form, with Δ*H*_α→β_ = –1.4 kJ mol^–1^ at 298 K. This temperature dependence is largely driven by a 3.1 kJ mol^–1^ stabilization of the β form arising from the lattice energies, which is partially canceled by the DFT phonon contribution (ESI Table S4†). The exothermic phase transition at higher temperatures predicted by MP2D is consistent with experiment. Linearly extrapolating Δ*H*_α→β_ to 423 K gives –2.0 kJ mol^–1^, in excellent agreement with both experimental values of –1.9 and –2.9 kJ mol^–1^ (Fig. 3).

Furthermore, MP2D Gibbs free energies indicate that the β form is thermodynamically preferred at elevated temperatures (Fig. 4b), which is consistent with the irreversible α → β transition seen experimentally at 423 K. Nominally, the MP2D calculations predict an enantiotropic relationship between the two forms, though the 0.2 kJ mol^–1^ free energy difference between the two forms at 0 K is likely smaller than the inherent uncertainties in the models. The MP2D free energies suggest that the experimentally observed α → β phase transition at 423 K corresponds to a kinetically activated transformation from the metastable α form to the stable β one, rather than a true thermodynamic phase boundary.

To understand why MP2D performs well in this system while B86bPBE-XDM does not, Table 1 decomposes the lattice energy differences between the two polymorphs into their intra- and intermolecular contributions. MP2D and B86bPBE-XDM actually predict similar intermolecular energies that differ by only 0.3 kJ mol^–1^ for the fully relaxed 0 K structure, and by 1.3 kJ mol^–1^ for the room-temperature structures. Rather, the erroneous DFT predictions arise almost entirely from the intramolecular conformational energies: B86bPBE-XDM over-stabilizes the intramolecular hydrogen bond conformation by ∼6 kJ mol^–1^ (11% error) compared to gas-phase CCSD(T) benchmarks. Delocalization error is known to cause GGA and hybrid functionals to overstabilize aromatic systems.^24^,^131^,^132^ The intramolecular conformation in the β polymorph disrupts not only the intramolecular hydrogen bond, but also π conjugation between the aromatic ring and the amide/acetamide side chains. In contrast to B86bPBE-XDM, MP2D reproduces the CCSD(T) conformational energy difference to within a few tenths of a kJ mol^–1^ (<1% error).

Finally, the *o*-acetamidobenzamide polymorphs demonstrate the importance of correcting both the intra- and intermolecular description of dispersion in MP2, as is done in MP2D. MP2 underestimates the intermolecular preference for the β phase by 4–5 kJ mol^–1^, and it underestimates the penalty for disrupting the intramolecular hydrogen bond found in the α form by 2 kJ mol^–1^ (Table 1). These errors cancel somewhat, but the resulting Δ*H* and Δ*G* values appear to change too rapidly with temperature (due to how the lattice energy varies with the temperature-dependent changes in crystal structure). MP2C corrects the description of the intermolecular interactions, but it does not alter the intramolecular description. This disrupts the fortuitous error cancellation found in MP2, and MP2C overestimates the stability of the β form substantially.

### 3.2 ROY

**Fig. 5:**
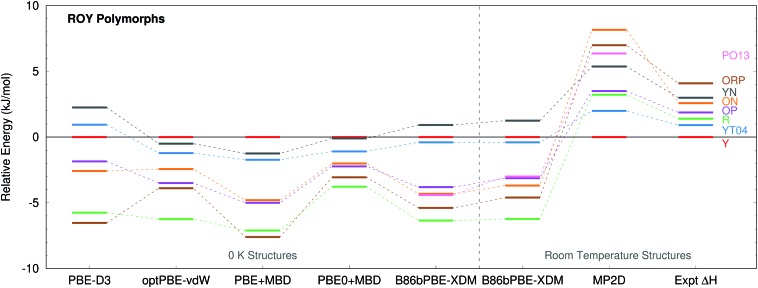
Comparison between predicted lattice energies and experimentally measured enthalpies^86^ for 8 polymorphs of ROY, all relative to form Y. The PBE-D3, optPBE-vdW, PBE + MBD, and PBE0 + MBD results were taken from ref. 41. They omit PO13 and use fully relaxed 0 K unit cells. The other results employ fixed-cell room-temperature structures.

ROY is among the most prolific conformational polymorph formers known. Seven polymorphs have been well-characterized for years.^86^,^106^,^107^ Since 2018, the structures of two more polymorphs, R05 41 and PO13,^108^ have been solved, and a structure for the RPL polymorph was proposed.^42^ However, as multiple recent studies have noted, predicting the energetics of these polymorphs has proved challenging.^41^,^42^,^133^ Many well-regarded van der Waals-inclusive GGA and hybrid density functionals predict highly incorrect polymorph orderings, including PBE-D3, PBE-NP, optPBE-vdW, PBE + MBD, and PBE0 + MBD (Fig. 5).^41^,^42^ Most strikingly, the DFT calculations frequently suggest that the Y polymorph is one of the least stable forms, when it is actually the most stable polymorph experimentally. Inclusion of zero-point energies or thermal contributions does not correct the rankings, either.^42^

Experimentally, the relative free energies of the ROY polymorphs were measured in the ∼40–120 °C range by eutectic melting experiments.^106^,^107^,^134^,^135^ Relative enthalpies were then obtained from the slopes of Δ*G*/*T vs.* 1/*T* plots, where *T* is temperature. Because the fitted enthalpies lack explicit temperature dependence, they are most valid in the elevated temperature regime under which they were measured. To facilitate comparison against the experimental data, fixed-cell room-temperature crystal structures are used for the polymorph stability calculations here, since they will mimic the structures under the experimental conditions better than fully relaxed 0 K ones.

B86bPBE-XDM calculations on the room-temperature structures predict stabilities that are similar to earlier DFT studies (Fig. 5), with the Y form being the second least-stable polymorph in terms of lattice energy (below only the YN form). Differences in the polymorph stabilities between the fixed-cell room-temperature and fully-optimized 0 K structures are modest. Refining the lattice energies of the room-temperature structures with single-point energy calculations at the fragment-based MP2D level completely transforms the crystal energy landscape. MP2D correctly predicts the Y form to be the most stable in terms of lattice energy. Furthermore, with the exception of the ON polymorph, the MP2D stability ordering qualitatively matches the experimental enthalpy data perfectly. The predicted lattice energy differences are somewhat larger than the experimental enthalpies. That discrepancy may in part be due to the omission of phonon contributions in the predictions here. The reason for the incorrect ordering of the ON polymorph is unclear. While an earlier study using a different density functional had difficulty reproducing the experimental ON crystal structure, the structure obtained here with B86bPBE-XDM agrees well with experiment (ESI Section S1.1†).

The MP2D calculations also predict the recently discovered PO13 polymorph to be among the less stable polymorphs, below only ON and ORP. Though experimental thermochemical data is not available for the PO13 form, this prediction is consistent with experimental data that indicates PO13 is less stable than the Y polymorph and that its heat of fusion is in the mid-range compared to the other forms. Predictions for the R05 form are omitted here because, unlike the other polymorphs, its cell has a net dipole which creates difficulties for the fragment-based approach (ESI Section S3.4†).

**Fig. 6:**
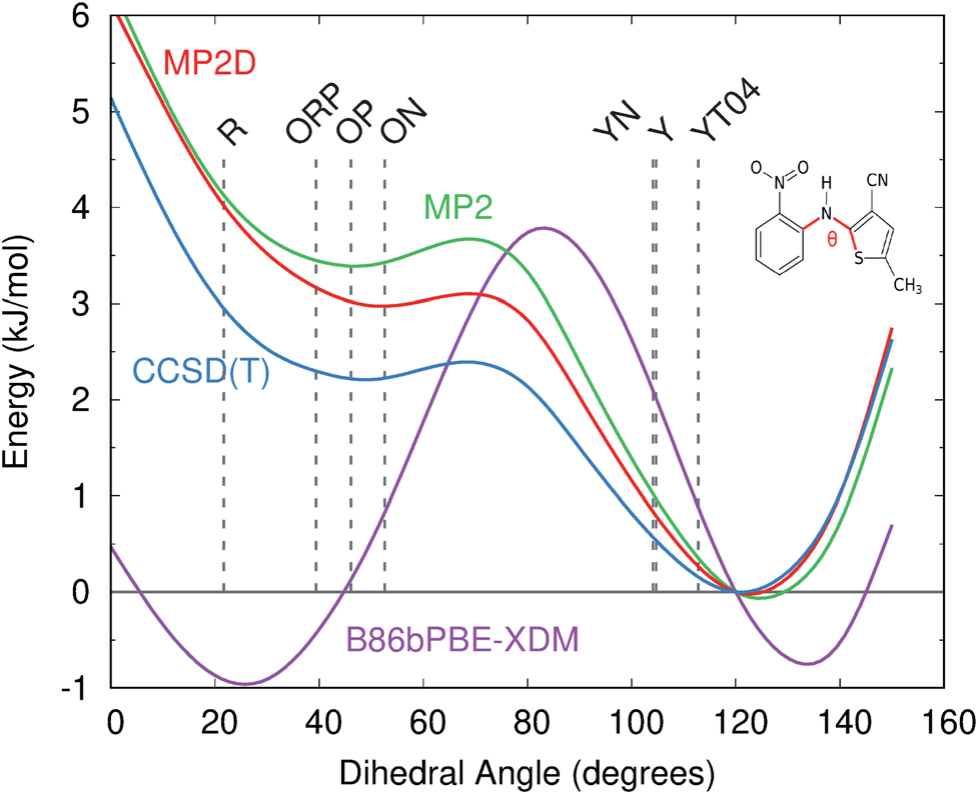
Comparison of the intramolecular conformational energy scan for the key intramolecular dihedral angle in ROY at several levels of theory. Vertical dotted lines indicate the experimental dihedral angles for each polymorph. Energies are relative to the 120° conformation, which corresponds to the CCSD(T) minimum.

Like for *o*-acetamidobenzamide, the major differences between MP2D and B86bPBE-XDM for the ROY polymorph energies stem from the problematic B86bPBE-XDM intramolecular treatment of the conformational energies. Fig. 6 plots the one-dimensional conformational energy scan along a key dihedral angle associated with the different conformations found in the ROY polymorphs. For convenience, vertical lines in the figure highlight the corresponding values of this dihedral angle found in the different polymorphs (though the other degrees of freedom along this scan may differ from those found in the actual crystals). Similar conformational energy scans can be found in earlier studies,^42^,^133^ albeit without the CCSD(T) benchmarks provided here. Compared to CCSD(T), B86bPBE-XDM dramatically overstabilizes the conformations found in the red (R) and orange (O) polymorphs relative to those occuring in the yellow (Y) forms. This explains why the earlier DFT calculations predict the yellow forms to be so much less stable than the others. In contrast, MP2D mimics the CCSD(T) conformational energy profile much more faithfully. MP2D does underestimate the stability of the conformations with the dihedral angles adopted by the red and orange forms by up to ∼1 kJ mol^–1^ relative to CCSD(T). Nevertheless, MP2D represents a substantial improvement over B86bPBE-XDM. The intramolecular MP2D dispersion correction improves the MP2 conformational energies modestly, by up to ∼1 kJ mol^–1^ (Fig. 6). At the same time, the MP2D dispersion correction does not alter the qualitative MP2 polymorph stability ordering in this system (ESI Section S3.2†).

**Fig. 7:**
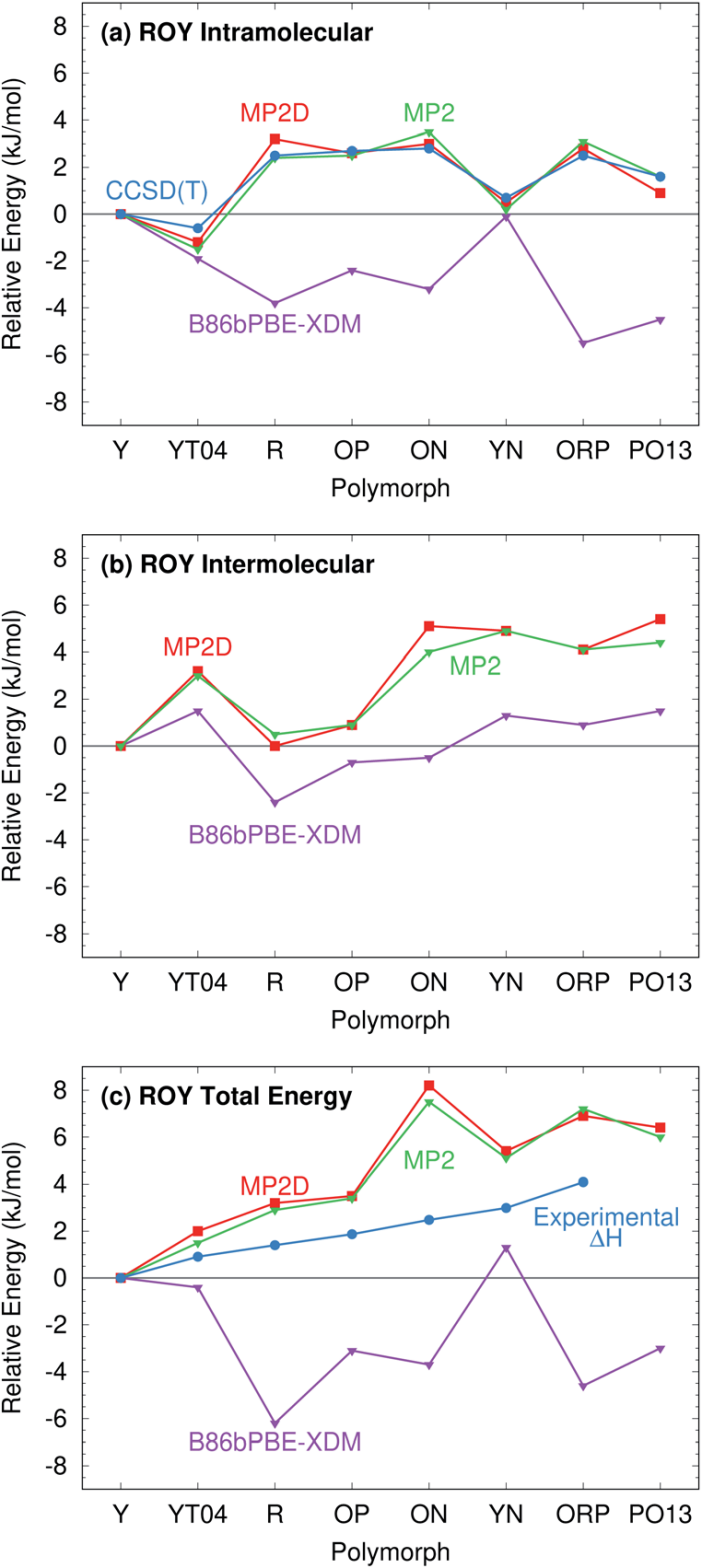
Energy decomposition of the room-temperature structure ROY polymorph lattice energies into (a) intramolecular, (b) intermolecular, and (c) total energy contributions. The energies are plotted relative to the most stable Y polymorph in each case.

For further insight, Fig. 7 decomposes the relative polymorph energies into their intra- and intermolecular contributions. As expected from the one-dimensional conformational energy scan, B86bPBE-XDM overstabilizes the intramolecular conformations of the orange and red polymorphs, while MP2 and MP2D give conformational energies which are quite faithful to CCSD(T). The intermolecular trends plotted in Fig. 7b are qualitatively similar between the three models. Indeed, the intermolecular energies are roughly parallel between methods for most of the polymorphs. The most notable difference is that B86bPBE-XDM appears to stabilize the intermolecular interactions of the other forms relative to Y more so than do MP2 and MP2D, which shifts all points other than Y in the B86bPBE-XDM curve down relative to the MP2-based ones in Fig. 7b. Unfortunately, coupled cluster benchmarks that could assess the quality of the two models for the intermolecular interactions are computationally infeasible.

Combining the intra- and intermolecular contributions (Fig. 7c), one sees once again that the MP2 and MP2D curves are in much better agreement with experiment than the B86bPBE-XDM one is. The MP2D energy of the ON polymorph is the most notable outlier relative to experiment. The fact that MP2D predicts the intramolecular conformational energy of the ON polymorph to within 0.2 kJ mol^–1^ of CCSD(T) suggests that any problem in the predicted energy ranking arises from the intermolecular contributions.

### 3.3 Oxalyl dihydrazide

**Fig. 8:**
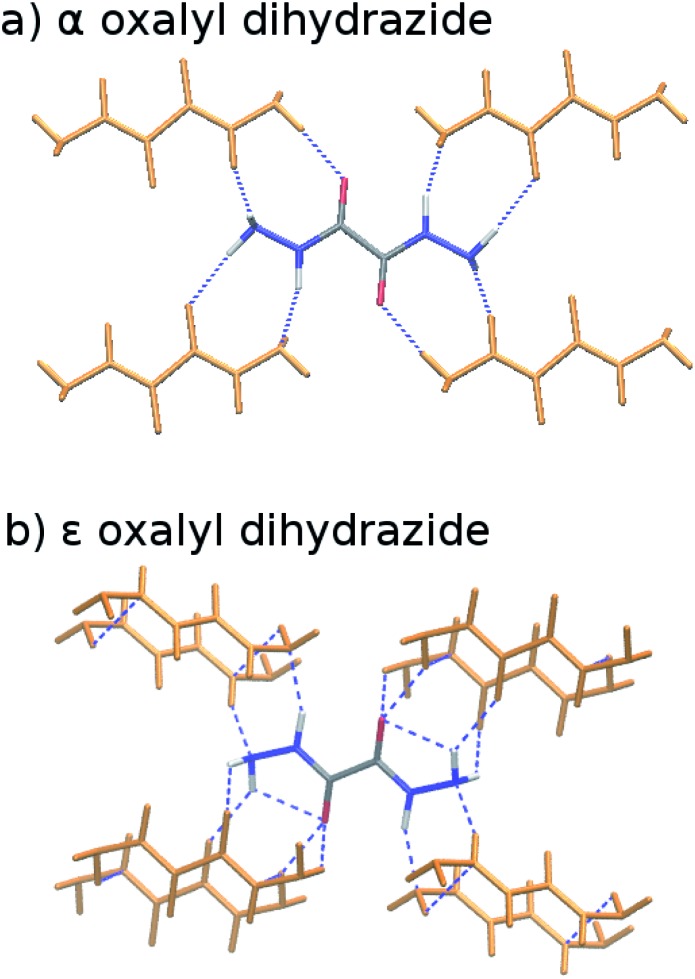
Crystalline oxalyl dihydrazide exhibits (a) purely intermolecular hydrogen bonding in the α polymorph and (b) a mixture of intra- and intermolecular hydrogen bonding in the other four polymorphs (ε form shown here).

The five polymorphs of oxalyl dihydrazide differ in whether the crystal packing contains purely intermolecular hydrogen bonding (α form) or exhibits a mixture of both intra- and intermolecular hydrogen bonds (β, γ, δ, and ε forms), as shown in Fig. 8.^87^ Five additional high-pressure polymorphs have been reported,^136^ but their structures are unknown and they are not considered here. Experimentally, the α, ε, and δ forms are the most stable polymorphs, though the ranking among those three is uncertain. The α form should arguably be the most stable polymorph based on its high density,^87^ but exceptions to density-based stability arguments can occur in hydrogen bonded crystals. All three forms convert endothermically to γ near 200–210 °C. This suggests that the γ form has a lower free energy at these temperatures, but that the α, ε, and δ forms have lower enthalpies.^87^ This indicates an enantiotropic relationship between γ and the other three forms according to the heat-of-transition rule.^137^ Finally, the β form has proved difficult to produce and characterize, and it converts readily to the α form.^87^,^136^ Therefore, it is assumed to be the least stable form. Overall, the inferred lattice energy ranking is (from most to least stable): α, δ, ε < γ < β.

These five polymorphs have been studied theoretically by several groups,^10^,^40^,^58^,^123^ and the results have been summarized by a couple authors.^8^,^10^ Initial DFT calculations involving empirical dispersion corrections predicted lattice energies consistent with the aforementioned stability ordering, but the lattice energies spanned a surprisingly large range of ∼15 kJ mol^–1^.^40^ Subsequent DFT calculations employing more modern dispersion corrections narrowed the polymorph energy range modestly to ∼10–12 kJ mol^–1^. Prior HMBI fragment-based MP2 and MP2C calculations also achieved the same stability ordering, albeit with much smaller ∼3–4 kJ mol^–1^ energy window.^123^ That study found that the competition between intra- and intermolecular basis set superposition error plays a substantial role in the energetics and that large basis sets are needed when atom-centered basis functions are used. Fully periodic local MP2 calculations performed two years later found a ∼10 kJ mol^–1^ energy range for the polymorphs,^58^ which are consistent with the DFT calculations, though the double-zeta basis set is probably too small to draw firm conclusions.

**Fig. 9:**
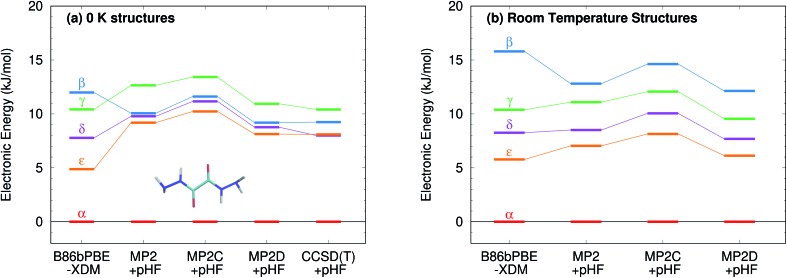
Relative stabilities of the oxalyl dihydrazide polymorphs at several levels of theory using both the fully relaxed 0 K structures and the fixed-cell room-temperature structures. For the MP2 methods, the relative stabilities of the β and γ forms differ depending on which structure optimization is used.

Fig. 9 presents new B86bPBE-XDM DFT results and HMBI-based MP2, MP2D, and MP2C single-point energy refinements of those DFT structures. For the fully relaxed 0 K structures, all four methods exhibit energy gaps in the ∼10–12 kJ mol^–1^ energy range, consistent with the earlier DFT and periodic local MP2 calculations. The HMBI results here should be more reliable than the previously published ones,^123^ since the geometries were optimized with a more robust B86bPBE-XDM dispersion-corrected DFT functional and because the many-body terms are evaluated with periodic HF instead of a polarizable force field. The strong, favorable polarization that is found only in the α polymorph makes the energy gap between the α and other four forms sensitive to the many-body description. The small energy range for the polymorphs predicted in ref. 123 appears to be an erroneous artifact of the polarizable force field contributions used there.

Like earlier calculations, the relative B86bPBE-XDM lattice energies for the fully relaxed 0 K structures are consistent with the inferred experimental lattice energy stability ordering: α < δ < ε < γ < β. Using the same structures, the MP2-based methods agree that α < δ < ε < γ, though the ε form is somewhat less stable than it is with DFT. However, MP2-based methods predict that the β form is more stable than the γ one, contrary to what has been inferred experimentally. Fragment CCSD(T) calculations similarly predict the β form to be more stable. They also predict the δ form to be marginally (0.1 kJ mol^–1^) more stable than ε. As noted earlier, the experimental stability ranking among α, δ, and ε is unclear.

Because the limited experimental knowledge of the β form is based on its instability at ambient conditions, the calculations were repeated using the fixed-cell room-temperature crystal structures. Using these structures destabilizes the lattice energy of the β polymorph relative to the α one by 4 kJ mol^–1^ with B86bPBE-XDM, and by 2–2.5 kJ mol^–1^ for the MP2-based methods (Fig. 9). Moreover, the MP2 models stabilize the room-temperature γ, δ, and ε structures by 1–2 kJ mol^–1^ relative to the α one. The end result is that all methods predict the α < δ < ε < γ < β stability ordering when room-temperature structures are used.

**Fig. 10:**
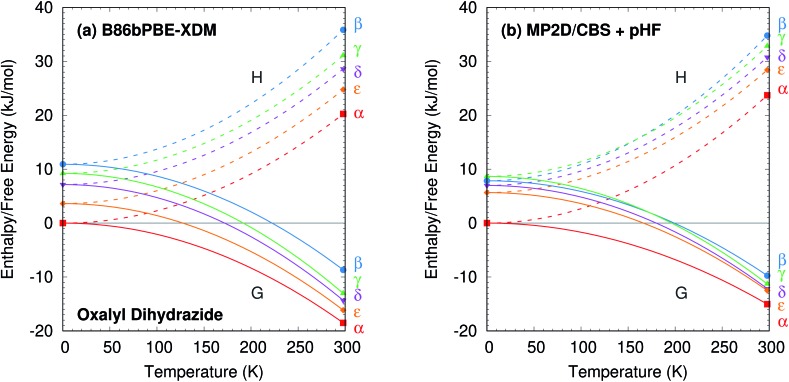
Relative enthalpy *H* and free energy *G* curves for the five polymorphs of oxalyl dihydrazide computed using (a) B86bPBE-XDM or (b) fragment-based MP2/CBS + pHF. The most notable difference between the two curves is that the β and γ forms are monotropically related in the DFT calculations, but enantiotropically related with MP2D (the *G* curves intersect near 175 K). The data points indicate computed results, while the curves connecting them are schematic.

The predicted temperature dependence translates directly to the enthalpies and free energies shown in Fig. 10. Augmenting the electronic energies with phonon contributions stabilizes the β, γ, δ, and ε polymorphs relative to α, but it does not alter the predicted stability orderings for the DFT or MP2 models (ESI Table S7 and Fig. S7†).

The difference between the lattice energies and free energies is interesting. First, the free energies span a much narrower range than the lattice energies—*e.g.* 6 kJ mol^–1^*versus* 15 kJ mol^–1^ for MP2D at 298 K—indicating the importance of entropic contributions in this system. Second, the free energy of the γ form stabilizes more rapidly with increasing temperature than do those of the α, δ, and ε forms. That is consistent with the experimental evidence for the γ form being enantiotropically related to the other three polymorphs.

Overall, the DFT and MP2-based results here are all consistent with available experimental observations. More experimental data would be needed to discriminate between the distinct DFT and MP2-based predictions for the β and γ polymorph stabilities. However, two points can be made based on currently available information. First, the experimental crystal structure of β has greater uncertainty than the other forms.^87^ That could affect the β fixed-cell room-temperature structure moreso than the fully relaxed one. Notably, the β form contracts ∼8% upon full geometry optimization, compared to only ∼4% for the other four polymorphs. This bigger structural change between 0 K and room-temperature manifests in the correspondingly large lattice energy change seen for the β form. Of course, this larger structural change in the β form might also simply reflect differences in the crystal packing that increase its thermal expansivity.

**Fig. 11:**
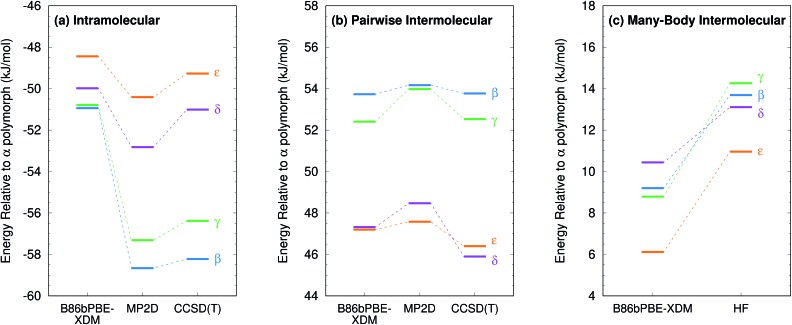
Energy decomposition for the oxalyl dihydrazide polymorph stabilities into (a) intramolecular (1-body), (b) pairwise intermolecular (2-body), and (c) many-body intermolecular contributions. Note, in the HMBI fragment approach, both MP2D and CCSD(T) employ the same HF many-body treatment. The same 14 kJ mol^–1^ energy range is used in all three figures to facilitate comparisons.

Second, energy decomposition and CCSD(T) benchmarks on the fully-relaxed structures in Fig. 11 suggest that the MP2D energetics are more accurate than those from B86bPBE-XDM. MP2D and CCSD(T) both predict the intramolecular conformations found in the β and γ polymorphs to be considerably more stable than those adopted in the δ and ε polymorphs. In contrast, B86bPBE-XDM predicts erroneously small energy differences between the conformations. For the total pairwise intermolecular interactions, B86bPBE-XDM and MP2D predict fairly similar energetics, with the DFT results actually agreeing better with CCSD(T). This is due to fortuitous error cancellation: examining all the individual dimer interactions, MP2D exhibits a root-mean-square (rms) error of 0.4 kJ mol^–1^*versus* CCSD(T), compared to 0.8 kJ mol^–1^ for B86bPBE-XDM. Similarly, looking at total two-body contributions instead of relative lattice energies, the B86bPBE-XDM 2-body contributions are systematically under-bound by rms error 7.3 kJ mol^–1^ compared to CCSD(T). In contrast, MP2D overbinds by a much smaller rms 1.6 kJ mol^–1^. In other words, the B86bPBE-XDM 2-body terms have much larger errors, but they exhibit better systematic error cancellation here to produce the agreement seen in Fig. 11b.

The other major difference between models occurs for the many-body contributions. The many-body contributions in all three systems explored in this paper generally amount to only ∼5% of the total lattice energy (and always less than 10%), which is typical for organic molecular crystals.^114^ However, because the many-body contributions in a system like oxalyl dihydrazide vary considerably between polymorphs, they play an out-sized role in determining the relative lattice energies. As shown in Fig. 11c, the relative many-body contributions in oxalyl dihydrazide have the same magnitude as the overall lattice energy differences. Compared to B86bPBE-XDM, periodic HF predicts a considerably stronger polarization effect for the α form that effectively shifts the relative energies of the other polymorphs up. Furthermore, HF predicts the β and γ forms to have more repulsive many-body contributions than does B86bPBE-XDM. While experimental or higher-level theoretical benchmarks are not available to determine which many-body treatment is more accurate, it is clear that B86bPBE-XDM obtains the correct experimental stability ordering only by error cancellation between the intramolecular and many-body intermolecular contributions. For example, if one corrected the erroneous intramolecular B86bPBE-XDM conformational energies with CCSD(T) ones, the result would incorrectly predict that the δ form is the least stable polymorph by 2 kJ mol^–1^. Obtaining the proper stability ordering would also require replacing the B86bPBE-XDM many-body energies with values similar to those obtained from periodic HF. This suggests that the HF many-body contributions are likely closer to the true values.

Finally, as shown in ESI Table S8,† the MP2-based methods all predict the 1-body energies with similar accuracy. However, for the 2-body interaction energies, MP2 and MP2C systematically overbind the dimers with rms errors of 8.1 kJ mol^–1^ and 4.2 kJ mol^–1^, respectively, both of which are several-fold larger than the 1.6 kJ mol^–1^ error for MP2D (also mildly over-bound). In other words, the similar relative polymorph stabilities seen for the different MP2-based methods in Fig. 9 arise from systematic cancellation of the overbinding errors that occur in MP2 and MP2C.

## 4 Conclusions

For many years, it has been widely recognized that balancing intra- and intermolecular conformational energies is one of the primary obstacles to crystal structure prediction in conformational polymorphs. Since the widespread adoption of DFT in crystal structure prediction, however, this issue of balancing the intra- and intermolecular interactions has been given much less attention. While periodic DFT models may have reduced the prevalence of such balance issues compared to earlier force field studies, the results here clearly demonstrate that widely used density functionals have not yet solved the problem of ranking conformational polymorphs in crystal structure prediction.

This study examined three well-known and challenging examples of conformational polymorphism: *o*-acetamidobenzamide, ROY, and oxalyl dihydrazide. In the first two systems, a variety of dispersion-corrected DFT models predict catastrophically wrong relative polymorph stabilities. The ∼8 kJ mol^–1^ DFT errors in relative polymorph stabilities found in these two systems greatly exceed the few kJ mol^–1^ error often associated with DFT polymorph rankings. More importantly, given that over 95% of polymorph pairs exhibit energy differences less than 8 kJ mol^–1^, and the majority have energy differences less than 2 kJ mol^–1^, such errors are unacceptable in crystal structure prediction. In both systems, the problem for DFT arises largely from a poor description of the intramolecular conformational energy.

The third system, oxalyl dihydrazide, is more nuanced, in part due to greater ambiguity in the experimental data. High-quality experimental thermochemical measurements of this (and other) polymorphic systems would be valuable for assessing the performance of different models more clearly. Based on presently available data, the overall DFT energy rankings for the oxalyl dihydrazide polymorphs do appear generally consistent with experiment. However, energy decomposition and coupled cluster theory benchmarks suggest that such nominal agreement arises from substantial cancellation of errors between the intra- and intermolecular interactions.

While the present study focused on the B86bPBE-XDM functional, it is important to recognize that these issues transcend any individual density functional. They occur for both GGA and hybrid density functionals with a variety of dispersion treatments, as evidenced by Fig. 5. Furthermore, the conformational polymorph examples here raise the question: How common are such failures of DFT for crystal structure prediction? Are these three systems outliers? Will more examples be uncovered as DFT-based crystal structure prediction techniques are increasingly applied to larger, more flexible pharmaceutical molecules?

This work also demonstrates that fragment-based MP2D calculations provide a promising path forward. For ROY and *o*-acetamidobenzamide, the higher-level calculations not only correct the qualitative polymorph stability ordering, but they also predict relative stabilities that are in generally good agreement with experiment. For oxalyl dihydrazide, MP2D also appears to perform better than DFT based on the energy decomposition results, though more experimental data to confirm the predictions would be helpful.

The study also examined a few variants of MP2. The balanced description of both intra- and intermolecular dispersion makes MP2D superior to MP2C, which corrects intermolecular dispersion only, and to MP2, which performs poorly for both intra- and intermolecular dispersion. The intramolecular dispersion correction contributes in all three systems, but it proves critical in predicting the stability for the *o*-acetamidobenzamide polymorphs. Fortunately, the MP2D dispersion correction can be computed with trivial effort once MP2 results are available.

The overall computational cost of these calculations is considerably higher than DFT, with complete-basis-set MP2D single-point energies requiring approximately 900, 7000, and 21 000 central processing unit (CPU) hours per polymorph of oxalyl dihydrazide (C_2_H_6_N_4_O_2_), acetamidobenzamide (C_9_H_10_N_2_O_2_), and ROY (C_12_H_9_N_3_O_2_S). This computational cost is dominated by the evaluation of ∼40–50 dimer interactions at the large-basis MP2D limit and the *N*^5^ MP2 scaling with monomer/dimer size.^10^ Fortunately, the number of monomer and dimer fragments scales linearly with increasing number of molecules in the asymmetric unit.^79^,^113^ Furthermore, those fragment calculations can be run independently and in parallel, making it feasible to perform crystalline calculations in much shorter amounts of wall time in modern high performance computing environments. The present study demonstrates that such correlated wavefunction calculations are feasible in species that are comparable in size to small-molecule pharmaceuticals. Efforts are currently underway to improve the accuracy and reduce the computational costs of these correlated wavefunction models further.

## Conflicts of interest

There are no conflicts to declare.

## Supplementary Material

Supplementary information

Supplementary information
