# Supplementary material for: Overcoming the difficulties of predicting conformational polymorph energetics in molecular crystals via correlated wavefunction methods
Source: Chem Sci. 2020 Jan 14;11(8):2200–14. doi: 10.1039/c9sc05689k (PMC7059316; doi:10.1039/c9sc05689k)
Supplement: Supplementary file 1 [file SC-011-C9SC05689K-s001.pdf]

Electronic Supplementary Material (ESI) for Chemical Science.
This journal is © The Royal Society of Chemistry 2020

Supplementary Information for "Overcoming the difficulties of predicting conformational polymorph energetics in molecular crystals via correlated wavefunction methods"

Chandler Greenwell,[†] Jessica McKinley,[†] Peiyu Zhang,[‡] Qun Zeng,[‡] Guangxu Sun,[‡] Bochen Li,[‡] Shuhao Wen,[‡] and Gregory J.O. Beran[*,†]

†*Department of Chemistry, Univeristy of California, Riverside, California 92521 USA*

‡*Xtalpi, Inc. 245 Main St, 12th Floor, Cambridge MA 02142*

E-mail: gregory.beran@ucr.edu

Phone: +1 (951) 827-7869

Contents

S1 Additional Computational Details **3**
S1.1 DFT Monkhorst-Pack grids and quality of the optimized structures 3
S1.2 Basis set dependence for the MP2 models 6
S1.3 Selection of the Hartree-Fock many-body treatment basis set 8

S2 *o*-Acetamidobenzamide polymorphs **10**
S2.1 Enthalpies and Gibbs Free Energies 10

S3 ROY polymorphs **12**
S3.1 Relative polymorph energies 12
S3.2 Impact of the MP2D dispersion correction 13
S3.3 Comparison of 0 K and room-temperature structures 14
S3.4 Omission of the R05 polymorph 15

S4 Oxalyl Dihydrazide polymorphs **16**
S4.1 Energy decomposition of the polymorph contributions 18

References **20**

S1 Additional Computational Details

S1.1 DFT Monkhorst-Pack grids and quality of the optimized structures

The planewave DFT Monkhorst-Pack grids indicated in Table S1 were used for the B86bPBE-XDM geometry optimizations and single-point energies. These choices were made by systematically increasing the grid until the geometry no longer changed. Roughly speaking, 9 k-points were used for lattice constants below 4 Å, 7 k-points were used for lattice constants of 4–6 Å, 5 k-points for lattice constants of 6–8 Å, 3 k-points for lattice constants 8-12 Å, and 1 k-point for larger lattice constants. Table S1 also indicates the rmsd15 value for the 0 K (fully relaxed) and 298 K (fixed experimental lattice parameters) B86bPBE-XDM geometry optimizations. The rmsd15 metric indicates the root-mean-square deviation in the non-hydrogen atomic positions for a cluster of 15 molecules in each crystal.[1]

For *o*-acetamidobenzamide, the room-temperature x-ray diffraction structures ACBNZA and ACBNZA01 were chosen over the more recent low-temperature neutron diffraction structures (ACBNZA02 and ACBNZA03)[2] to enable examination of the temperature-dependent behaviors. Full B86bPBE-XDM relaxation of the neutron diffraction structures resulted in structures that were virtually identical to those used here (rmsd15 differences less than 0.04 Å and energy differences of 0.1 kJ/mol or less).

An earlier study[3] employing the PBE-NP dispersion-corrected DFT functional found that the ON structure relaxed considerably upon optimization, with the key intramolecular dihedral angle θ changing by $\sim$25°. This suggested that DFT might be particularly problematic for the ON polymorph. However, the B86bPBE-XDM functional used here does not appear to suffer from the same problems in reproducing the experimental crystal structures. For the fixed-cell optimization, the dihedral angle θ changes only 2.6°, which is on par with the average $3.4 \pm 2.4°$ change observed for the other seven polymorphs (Table S2). Relaxing the cell fully leads to a larger 9.5° change in that dihedral angle, but this is again on par with the average change of $8.2 \pm 3.6°$ change seen for the other forms. Structure overlays give rmsd15 values of 0.05 Å for the fixed cell and 0.22 Å for the fully relaxed cell relative to experiment, which are again compatible with values seen for the other crystals (Table S1). In other words, there is no obvious structural discrepancy between the B86bPBE-XDM and experimentally reported structures that might account for the disagreement in relative stabilities.

Table S1: Summary of **k**-point grids and quality of the agreement between the predicted and experimental crystal structures.

Crystal Polymorph	CSD RefCode	**k**-point grid	0 K Structure rmsd15 (Å)	298 K Structure rmsd15 (Å)
		***o*-Acetamidobenzamide**		
α form	ACBNZA	(7,3,3)	0.158	0.039
β form	ACBNZA01	(5,3,3)	0.144	0.050
		**ROY**		
form Y	QAXMEH01	(3,1,3)	0.220	0.048
form YT04	QAXMEH12	(5,3,3)	0.208	0.051
form R	QAXMEH02	(5,5,3)	0.163	0.052
form OP	QAXMEH03	(5,3,3)	0.199	0.075
form ON	QAXMEH	(9,1,1)	0.219	0.047
form YN	QAXMEH04	(7,3,3)	0.250	0.050
form ORP	QAXMEH05	(1,3,1)	0.454	0.053
form PO13	QAXMEH52	(7,1,3)	0.255	0.099
		**Oxalyl Dihydrazide**		
α form	VIPKIO01	(9,7,3)	0.071	0.040
β form	VIPKIO02	(9,3,7)	0.333	0.041
γ form	VIPKIO03	(7,1,5)	0.130	0.106
δ form	VIPKIO04	(9,1,7)	0.190	0.022
ϵ form	VIPKIO05	(7,9,3)	0.131	0.017

Table S2: Comparison of the experimental and predicted S-C-N-C dihedral angle θ for the different ROY polymorphs. Absolute errors between theory and experiment are indicated in parentheses. The key ON polymorph results are highlighted in bold.

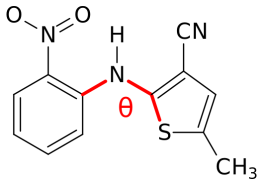

		B86bPBE-XDM		PBE-N2
	Experiment	298 K Structure	0 K Structure	0 K Structure
Y	104.7°	108.0° (3.2°)	112.5° (7.8°)	111.6° (6.8°)
YT04	112.8°	116.6° (3.7°)	119.8° (6.9°)	118.7° (5.8°)
R	21.7°	20.5° (1.2°)	19.5° (2.2°)	22.2° (0.4°)
OP	46.1°	43.0° (3.1°)	37.5° (8.6°)	40.1° (6.0°)
**ON**	**52.6°**	**50.0° (2.6°)**	**43.1° (9.5°)**	**26.2° (26.4°)**
YN	104.0°	104.4° (0.3°)	111.3° (7.3°)	112.3° (8.3°)
ORP	39.4°	34.4° (5.0°)	29.0° (10.4°)	31.0° (8.4°)
PO13	122.1°	129.5° (7.5°)	136.1° (14.0°)	135.3° (13.2°)

S1.2 Basis set dependence for the MP2 models

The HMBI calculations here rely on monomer and dimer MP2 calculations. MP2-based models exhibit slower convergence with basis set than do HF or DFT models, and the use of large basis sets is important for obtaining well-converged polymorph stability predictions. In this work, all MP2-based methods were extrapolated to the CBS limit from aug-cc-pVTZ and aug-cc-pVQZ results. Additionally, the MP2D dispersion correction was designed for use with large basis sets, and the short-range damping functions were fitted against CBS-limit results.[4]

Figures S1 and S2 show the dramatic impact of basis set completeness on the predicted polymorph stabilities for acetamidobenzamide and ROY. In acetamidobenzamide, the relative stability of the two forms changes by more than 6 kJ/mol between aug-cc-pVDZ and the CBS limit. Examination of the 1-body and 2-body contributions (not explicitly shown here) indicates that the basis set dependence is approximately evenly split between the intra- and intermolecular contributions. The ROY polymorph stabilities also exhibit strong basis set dependence. For polymorph ORP, the relative stability decreases 6.9 kJ/mol between aug-cc-pVDZ and the CBS limit. The average magnitude of the basis set change between aug-cc-pVDZ and the CBS limit is 3.6 kJ/mol, which again is substantial on the energy scale of polymorphism. The basis set dependence for oxalyl dihydrazide has been examined previously,[5,6] and similar results were found to those shown here for ROY and acetamidobenzamide.

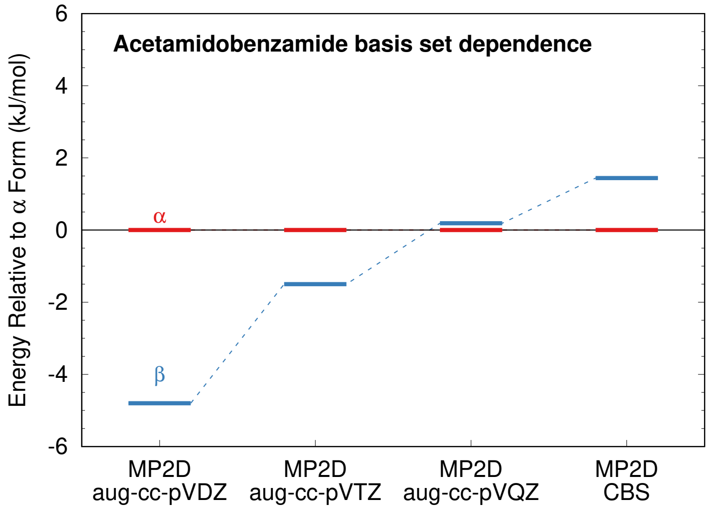

Figure S1: Dependence of the polymorph stabilities for *o*-acetamidobenzamide on the basis set used for the MP2D 1 & 2-body contributions. In all cases, periodic HF/pob-TZVP-rev2 was used for the many-body contribution.

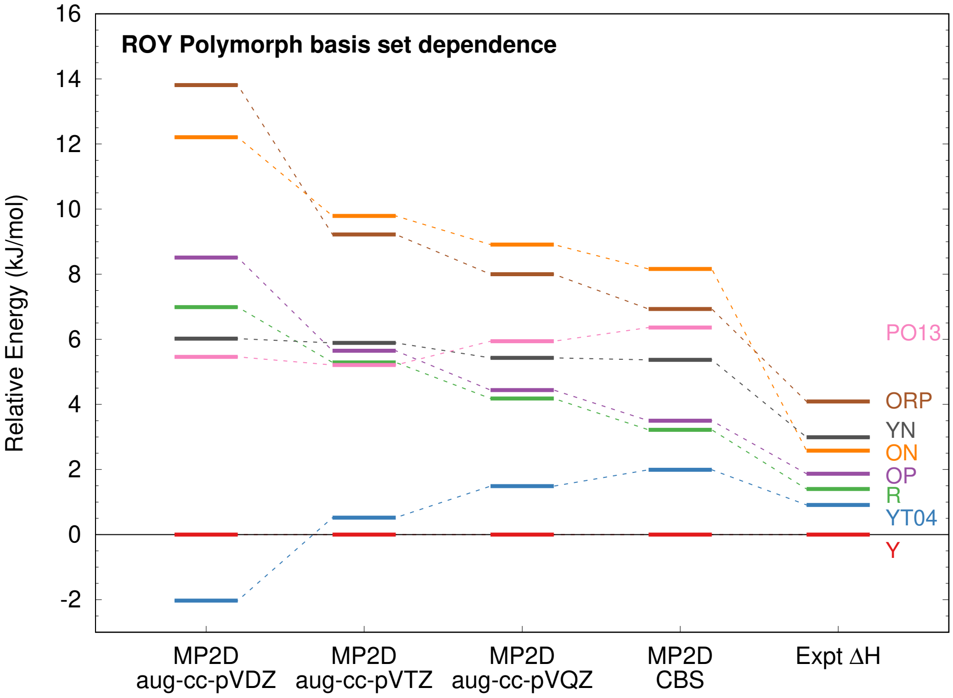

Figure S2: Dependence of the ROY polymorph stabilities on the basis set used for the MP2D 1 & 2-body contributions. In all cases, periodic HF/pob-TZVP-rev2 was used for the many-body contribution.

S1.3 Selection of the Hartree-Fock many-body treatment basis set

The 1-body (monomer) and 2-body (dimer) terms frequently account for 90% or more of the total lattice energy in the HMBI model, while the remaining ∼10% results from the intermolecular many-body contributions. However, in the polymorphic systems considered in this paper, the proportional impact of the many-body term on the relative polymorph energies is considerably larger. Therefore, it is important to obtain a well-converged description of the many-body term. Periodic HF is relatively inexpensive, particularly when Gaussian basis sets are employed, and can describe the frequently dominant many-body polarization effects.

Periodic HF (and DFT) calculations with large Gaussian basis sets suffer from well-known issues with linear dependencies and poor convergence of the self-consistent-field equations. Adapting a Gaussian basis sets designed for molecular systems to periodic calculations typically requires eliminating the most diffuse basis functions and subsequent re-tuning of the remaining basis functions. Several triple-ζ basis sets have been developed in recent years, including the pob-TZVP,[7] pob-TZVP-rev2,[8] and mTZVP basis set.[9] All three are modified versions of the popular def2-TZVP basis intended for periodic systems.

To assess the performance of these three basis sets, 15 five-molecule clusters were extracted from the oxalyl dihydrazide polymorphs. HF calculations were performed on the entire gas-phase cluster, and then separately subtracting out the HF monomer and dimer contributions according to a many-body expansion. Consistent with the HMBI treatment of the many-body terms, no counterpoise correction is employed in either the full cluster nor the fragment calculations. The HF/def2-QZVP results are chosen as the benchmark, and these results are compared against the traditional def2-TZVP basis set and the three aforementioned triple-ζ basis sets that are suitable for periodic calculations.

As shown in Table S3, the def2-TZVP basis set gives the root-mean-square (RMS) error of 0.31 kJ/mol per monomer versus def2-QZVP. The pob-TZVP-rev2 basis revised the pob-TZVP basis in order to reduce the occurrence of basis set superposition error (BSSE). While BSSE is more prevalent in dimer interactions, the results here show that the pob-TZVP-rev2 basis gives considerably smaller errors (0.31 kJ/mol) than pob-TZVP (0.63 kJ/mol) relative to the benchmark def2-QZVP values. Finally, the mTZVP basis, which typically exhibits even less BSSE, predicts many-body contributions with errors intermediate between the two other basis sets. In the end, the pob-TZVP-rev2 basis was adopted in this study based on these benchmarks.

Table S3: Predicted HF many-body energies (in kJ/mol per monomer) and the root-mean-square (RMS) error versus the def2-QZVP basis set results for 15 different clusters cut from the five polymorphs of oxalyl dihydrazide.

Cluster	def2-QZVP	def2-TZVP	pob-TZVP	pob-TZVP-rev2	mTZVP
α cluster #1	-1.87	-1.60	-1.51	-1.68	-1.20
α cluster #2	-4.05	-3.92	-4.20	-4.33	-3.82
α cluster #3	0.15	0.38	0.73	0.43	0.41
β cluster #1	-0.09	0.00	0.09	0.05	0.07
β cluster #2	0.00	0.21	0.29	0.02	0.40
β cluster #3	-0.02	0.27	0.55	0.29	0.49
γ cluster #1	0.36	0.60	0.89	0.68	0.62
γ cluster #2	0.05	0.15	0.35	0.25	0.13
γ cluster #3	0.33	0.58	0.83	0.66	0.65
δ cluster #1	0.17	0.36	0.61	0.44	0.46
δ cluster #2	-0.08	-0.11	0.45	0.23	-0.26
δ cluster #3	-0.70	-0.59	-0.50	-0.61	-0.54
ϵ cluster #1	-0.23	0.03	-0.03	-0.13	0.11
ϵ cluster #2	0.38	0.39	0.50	0.43	0.22
ϵ cluster #3	0.80	0.95	1.22	1.03	0.82
**RMS Error**		**0.31**	**0.63**	**0.37**	**0.51**

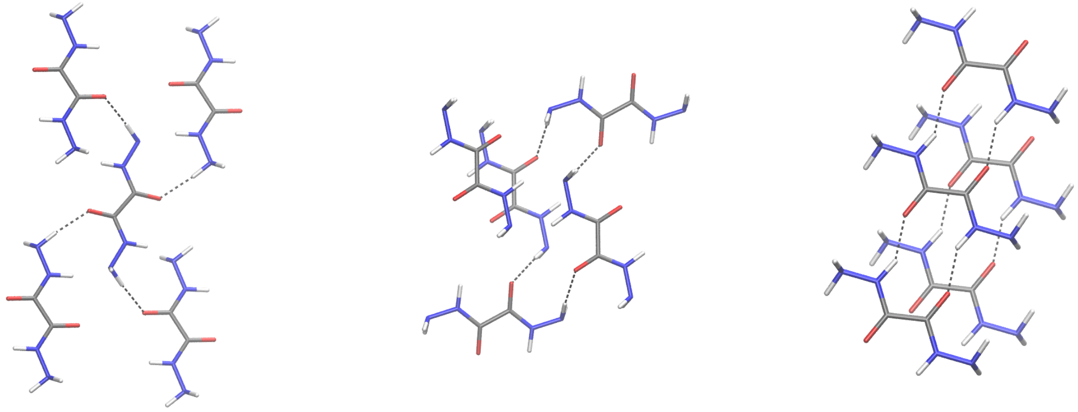

Figure S3: Three sample oxalyl dihydrazide pentamer clusters. From left to right: α cluster #2, β cluster #3, and δ cluster #2.

S2 *o*-Acetamidobenzamide polymorphs

S2.1 Enthalpies and Gibbs Free Energies

To augment the enthalpy and free energy curves shown in the main paper, Figure S4 plots the corresponding semi-schematic G and H curves for all four methods considered in the main paper. All three MP2-based models agree that the β form is thermodynamically preferred at high temperatures, though the specific free energies differ. MP2 predicts an enantiotropic relationship, while MP2C predicts a monotropic one. For MP2D, the relationship is also enantiotropic, though the two forms are nearly degenerate at 0 K. Consistent with experiment, all three MP2-based methods predict an exothermic $\Delta H_{\alpha\to\beta}$ phase transition at elevated temperatures. In contrast, B86bPBE-XDM incorrectly predicts an endothermic phase transition and that the α form is thermodynamically more stable at all temperatures. Examination of the $\Delta H_{\alpha\to\beta}$ values shown in Table 1 and Figure 3 of the main manuscript suggests that MP2C overestimates the stability of the β form due largely to the neglect of intramolecular dispersion.

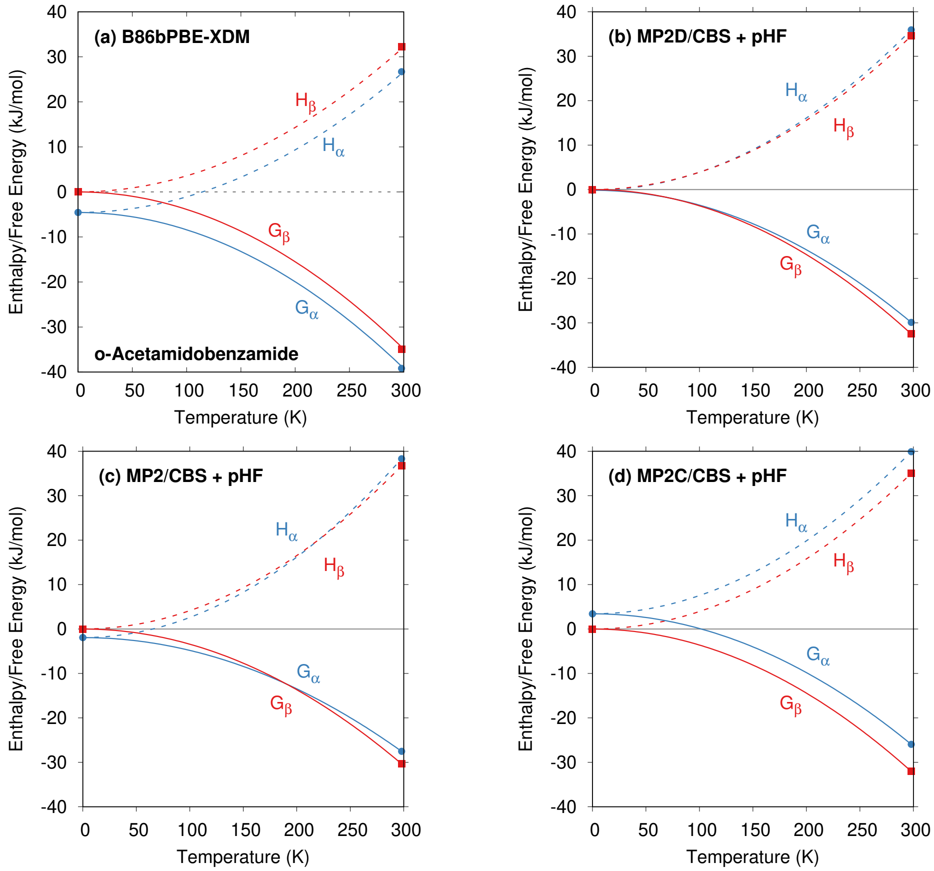

Figure S4: Comparison of the predicted enthalpies and Gibbs free energies for the two polymorphs of *o*-acetamidobenzamide as computed with several different models. Data points were computed, but the curves connecting them are merely schematic.

To understand the temperature dependence of the *o*-acetamidobenzamide predictions better, Table S4 decomposes the temperature dependence into lattice energy contributions (due to thermal expansion) and phonon contributions. Note that the MP2-type results use B86bPBE-XDM phonon frequencies, meaning that the vibrational contributions to the enthalpy H_{vib} and free energy G_{vib} are identical across all methods here.

For B86bPBE-XDM, the lattice energy changes stabilize the β form relative to α by less than 1 kJ/mol over the 0–423 K temperature range, while the vibrational enthalpy destabilizes the β form by 2.3 kJ/mol. The MP2-based models all predict more substantial lattice energy-driven stabilization of the β polymorph with increasing temperature, ranging from -7.3 kJ/mol for MP2 to -4.3–4.4 for the dispersion-corrected MP2D and MP2C models. These lattice energy changes favoring the β form at higher temperatures are partially canceled by the phonon contributions that increasingly favor the α form at higher temperatures, leading to the final predicted MP2D enthalpy that agrees very well with the experimentally measured transition enthalpy, as described in the main paper.

Table S4: Lattice energy differences, vibrational enthalpy contributions, and vibrational Gibbs free energy contributions to the α–β polymorph energy differences in *o*-acetamidobenzamide, in kJ/mol. Positive values indicate α is more stable than β. Summing the lattice energy and vibrational enthalpy contributions gives the results plotted in Figure 3 of the main paper.

	Lattice Energy Contributions				B86bPBE-XDM Phonon Contrib.	
T (K)	B86bPBE-XDM	MP2	MP2D	MP2C	H_{vib}	G_{vib}
0 K	5.8	3.2	1.4	-2.1	-1.3	-1.3
298 K	5.2	-1.9	-1.7	-5.2	0.3	-0.9
423 K[a]	4.9	-4.1	-3.0	-6.5	1.0	0.4
Change 0 K→298 K	-0.6	-5.1	-3.1	-3.0	1.6	0.4
Change 0 K→423 K	-0.9	-7.3	-4.4	-4.3	2.3	0.5

[a] Linearly extrapolated to 423 K from 0 K and 298 K values

S3 ROY polymorphs

S3.1 Relative polymorph energies

Table S5 summarizes the lattice energies computed here that were used to generate Figure 5 in the main paper.

Table S5: Summary of relative lattice energies (in kJ/mol) computed here using the fixed-cell room temperature crystal structures.

Polymorph	B86bPBE-XDM	MP2	MP2D
Y	0.0	0.0	0.0
YT04	-0.4	1.5	2.0
R	-6.2	2.9	3.2
OP	-3.1	3.4	3.5
ON	-3.7	7.5	8.2
YN	1.3	5.1	5.4
ORP	-4.6	7.2	6.9
PO13	-3.0	6.0	6.4

S3.2 Impact of the MP2D dispersion correction

Figure S5 compares HMBI results with MP2 and MP2D. The differences in the relative polymorph energies are less than 1 kJ/mol throughout. In all cases except ORP, the dispersion correction slightly destabilizes the polymorphs relative to Y. Note that although the impact on the relative polymorph energies is small in this instance, the dispersion correction has a big impact on the overall lattice energies. For example, MP2D reduces the strength of the 2-body interaction energies by 27–31 kJ/mol, depending on the polymorph.

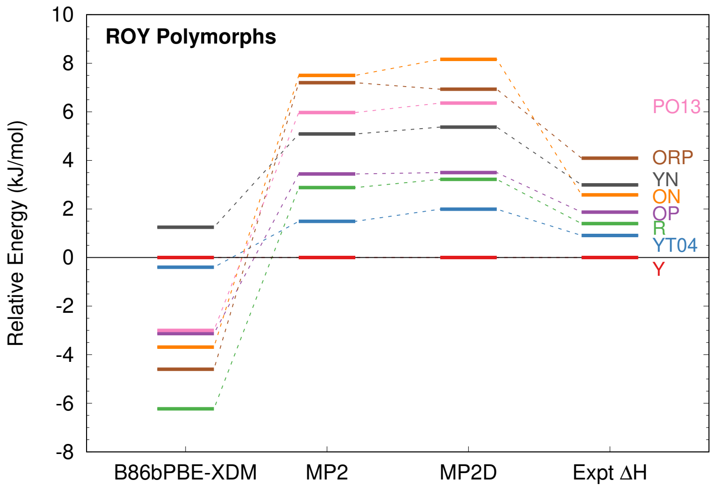

Figure S5: The impact of the MP2D dispersion correction on the relative polymorph energies is fairly modest in ROY, as compared to uncorrected MP2.

S3.3 Comparison of 0 K and room-temperature structures

Figure S6 plots the relative energies with DFT and MP2D for the fully relaxed 0 K structures and the fixed-cell room-temperature structures. Note that the results here compare MP2D in the aug-cc-pVTZ basis, rather than in the CBS limit like all other results presented here. This basis set was chosen for the 0 K structures to reduce computational costs.

At the DFT level, the differences in the relative lattice energies for the two sets of structures are quite small. For MP2D, they are considerably larger. The reasons behind the larger MP2D differences are not entirely clear. One potential explanation could be that the optimal MP2D geometries might differ somewhat from the DFT ones, and therefore the DFT optimal structures correspond to steeper portions of the MP2D potential energy surface (and therefore one obtains larger changes in the energies with the geometry changes).

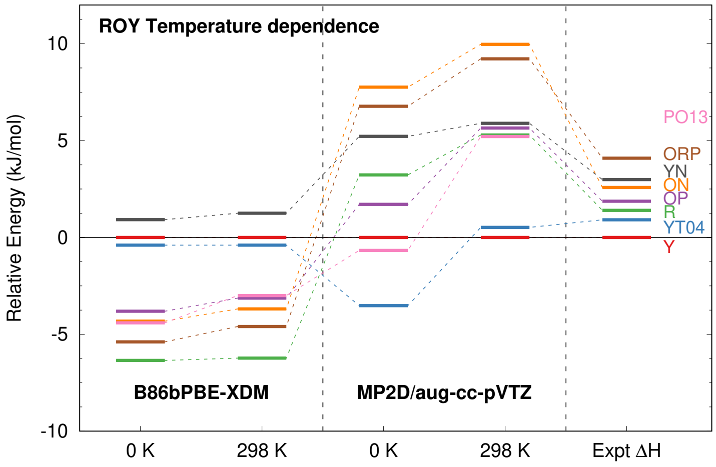

Figure S6: The impact of using fully-relaxed (0 K) structures versus fixed-cell relaxations (298 K) on the relative lattice energies.

S3.4 Omission of the R05 polymorph

The R05 polymorph is omitted from the present study because it was recently discovered that the HMBI model energies are poorly convergent in polar unit cells. The treatment of long-range electrostatics is generally problematic in periodic cells with a net dipole moment. In practice, this issue is frequently managed via application of tin-foil boundary conditions.

The working HMBI expression has the following form:

$$E^{HMBI} = E^{pHF}_{crystal} + \left(E^{MP2D}_{1body} - E^{HF}_{1body}\right) + \left(E^{MP2D}_{2body} - E^{HF}_{2body}\right) \tag{1}$$

where the 2-body terms are computed out to a cutoff radius of 9–10 Å. A smoothing function is applied across this interval to ensure smooth potential energy surfaces as the model transitions from the high-level (MP2D) to low-level (HF). The periodic HF contribution does employ tin-foil boundary conditions. In non-polar cells, one typically observes well-behaved convergence of the lattice energy as the 2-body cutoff radius is increased, as expected from the long-range decay of the interaction energies.

In polar unit cells, however, the contributions from the 2-body terms proves highly erratic with cutoff radius. For example, in γ-glycine, which has a polar unit cell, the energy relative to the non-polar α form can vary by many kJ/mol depending on the specific cutoff. For this reason, results on the R05 polymorph are omitted from the present study.

S4 Oxalyl Dihydrazide polymorphs

Table S6 presents the relative lattice energies for the oxalyl dihydrazide polymorphs using the 0 K and room-temperature structures—the same data used to generate Figure 9 in the main paper. For MP2 and CCSD(T), the many-body treatment is computed at the periodic HF/pob-TZVP-rev2 level of theory.

Table S6: Relative lattice energies (in kJ/mol) for the polymorphs of oxalyl dihydrazide using the fully relaxed 0 K structures or fixed-cell room-temperature structures.

	**0 K Structures**				
Polymorph	B86bPBE-XDM	MP2	MP2C	MP2D	CCSD(T)
α form	0.0	0.0	0.0	0.0	0.0
β form	12.0	10.1	11.6	9.2	9.2
γ form	10.4	12.7	13.4	10.9	10.4
δ form	7.8	9.8	11.2	8.8	8.0
ϵ form	4.9	9.2	10.2	8.1	8.1

	**298 K Structures**			
Polymorph	B86bPBE-XDM	MP2	MP2C	MP2D
α form	0.0	0.0	0.0	0.0
β form	15.8	12.8	14.6	12.1
γ form	10.4	11.1	12.1	9.5
δ form	8.3	8.5	10.0	7.7
ϵ form	5.8	7.0	8.1	6.1

Table S7 shows how the lattice energies, H_{vib} and G_{vib} of the different polymorphs change relative to the α form between the 0 K and room temperature structures. The lattice energy contributions here amount to the differences between the 0 K and 298 K results in Table S6. At the B86bPBE-XDM, the changes in the relative lattice energies are relative modest, except for the β form, which is destabilized by 3.8 kJ/mol. For the MP2-based methods, the β form is destabilized slightly less, by ∼3 kJ/mol. On the other hand, increasing temperature stabilizes the γ, δ, and ϵ forms by 1–2 kJ/mol relative to α. The temperature-dependence of the vibrational contributions to the enthalpy are relatively modest as well, with the other four forms being destabilized by 1–2 kJ/mol relative to the α form. In the end, the lattice energies and enthalpies predict the same qualitative polymorph stability orderings, with only small changes in the quantitative values, as can be seen from Figure S7. The key temperature-dependent change in the β/γ MP2D stability ordering arises from lattice energy contributions

Table S7: Change in the relative lattice energies, vibrational enthalpy contributions, and vibrational Gibbs free energy contributions of oxalyl dihydrazide polymorph stabilities between the 0 K and room-temperature structures, in kJ/mol. Values are listed relative to the α polymorph.

	Lattice Energy Contributions Change 0 K→298 K				B86bPBE-XDM Phonon Contrib. Change 0 K→298 K	
	B86bPBE-XDM	MP2	MP2D	MP2C	H_{vib}	G_{vib}
α form	0.0	0.0	0.0	0.0	0.0	0.0
β form	3.8	2.7	2.9	3.0	0.8	-5.0
γ form	0.0	-1.6	-1.4	-1.3	1.7	-3.7
δ form	0.5	-1.3	-1.1	-1.1	1.1	-3.0
ϵ form	0.9	-2.1	-2.0	-2.1	0.9	-1.3

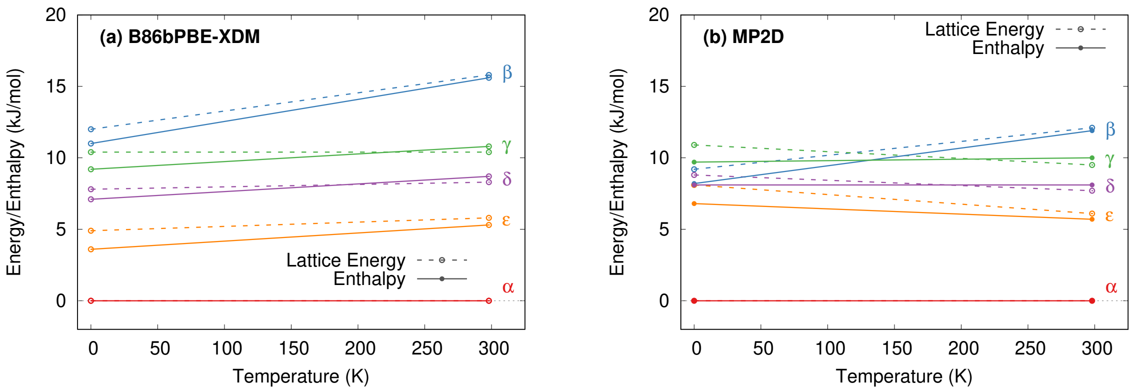

Figure S7: Temperature dependence of the relative lattice energies (dotted lines) and enthalpies (solid lines) for the oxalyl dihydrazide polymorphs. The qualitative stability ordering is unchanged for both lattice energies and enthalpies.

S4.1 Energy decomposition of the polymorph contributions

Table S8 shows energy decomposition data for oxalyl dihydrazide that was used to generate Figure 11 in the main paper. MP2 and MP2D give 1-body energies in much better agreement with the CCSD(T) benchmarks than does B86bPBE-XDM. For the two-body energies, B86bPBE-XDM does extremely well in the "relative" RMS error vs. CCSD(T) (i.e. as computed from the data shown in the table, where stabilities are specified relative to the α polymorph). However, if one instead computes the "absolute" RMS error based on the total 2-body interaction energies for each form (i.e. interaction energies relative to non-interacting monomers, summed over all dimers), the DFT functional performs far worse. MP2 performs even worse than B86bPBE-XDM, while MP2C does somewhat better on the absolute RMS error. MP2D reproduces CCSD(T) extremely well, with 1.6 kJ/mol errors in both the absolute and relative RMS errors.

Table S8: Decomposition of the relative polymorph electronic energies (kJ/mol) for oxalyl dihydrazide 0 K structures into intramolecular (1-body), pairwise intermolecular, and many-body intermolecular contributions. RMS errors are computed against the CCSD(T) benchmarks. All MP2 and CCSD(T) models employ the same periodic HF many-body treatment.

	**1-Body Intramolecular Contribution**			
Polymorph	B86bPBE-XDM	MP2	MP2D	CCSD(T)
α form	0.0	0.0	0.0	0.0
β form	-50.9	-58.5	-58.7	-58.2
γ form	-50.8	-57.2	-57.3	-56.4
δ form	-50.0	-52.8	-52.8	-51.0
ϵ form	-48.4	-50.4	-50.4	-49.3
RMS error	4.6	1.1	1.2	—

	**2-Body Intermolecular Contribution**				
Polymorph	B86bPBE-XDM	MP2	MP2C	MP2D	CCSD(T)
α form	0.0	0.0	0.0	0.0	0.0
β form	53.7	54.9	56.5	54.2	53.8
γ form	52.4	55.6	56.4	54.0	52.5
δ form	47.3	49.5	48.5	48.5	45.9
ϵ form	47.2	48.6	47.6	47.6	46.4
RMS error relative	0.8	2.7	3.8	1.6	—
RMS error absolute	7.3	8.1	4.2	1.6	—

	**Many-Body Contribution**	
	B86bPBE-XDM	HF
α form	0.0	0.0
β form	9.2	13.7
γ form	8.8	14.3
δ form	10.4	13.1
ϵ form	6.1	11.0

References

(1) Chisholm, J. A.; Motherwell, W. D. S. *J. Appl. Crystall.* **2005**, *38*, 228–231.

(2) Leech, C. K.; Barnett, S. A.; Shankland, K.; Gutmann, M.; Wilson, C. C. *Acta Cryst. B* **2006**, *62*, 926–930.

(3) Nyman, J.; Yu, L.; Reutzel-Edens, S. M. *CrystEngComm* **2019**, *21*, 2080–2088.

(4) Řezáč, J.; Greenwell, C.; Beran, G. J. O. *J. Chem. Theory Comput.* **2018**, *14*, 4711–4721.

(5) Wen, S.; Beran, G. J. O. *J. Chem. Theory Comput.* **2012**, *8*, 2698–2705.

(6) Beran, G. J. O. *Chem. Rev.* **2016**, *116*, 5567–5613.

(7) Peintinger, M. F.; Oliveira, D. V.; Bredow, T. *J. Comp. Chem.* **2013**, *34*, 451–459.

(8) Vilela Oliveira, D.; Laun, J.; Peintinger, M. F.; Bredow, T. *J. Comp. Chem.* **2019**, *40*, 2364–2376.

(9) Brandenburg, J. G.; Bannwarth, C.; Hansen, A.; Grimme, S. *The Journal of Chemical Physics* **2018**, *148*, 064104.
